# A review of the machine learning datasets in mammography, their adherence to the FAIR principles and the outlook for the future

**DOI:** 10.1038/s41597-023-02430-6

**Published:** 2023-09-08

**Authors:** Joe Logan, Paul J. Kennedy, Daniel Catchpoole

**Affiliations:** 1 Alixir Technologies Pty Ltd, Sydney, NSW Australia; 2 Australian Artificial Intelligence Institute, University of Technology Sydney, Sydney, NSW Australia; 3 The Tumour Bank, The Children’s Cancer Research Unit, Kids Research, The Children’s Hospital at Westmead, Sydney, NSW Australia

**Keywords:** Breast cancer, Developing world, Policy

## Abstract

The increasing rates of breast cancer, particularly in emerging economies, have led to interest in scalable deep learning-based solutions that improve the accuracy and cost-effectiveness of mammographic screening. However, such tools require large volumes of high-quality training data, which can be challenging to obtain. This paper combines the experience of an AI startup with an analysis of the FAIR principles of the eight available datasets. It demonstrates that the datasets vary considerably, particularly in their interoperability, as each dataset is skewed towards a particular clinical use-case. Additionally, the mix of digital captures and scanned film compounds the problem of variability, along with differences in licensing terms, ease of access, labelling reliability, and file formats. Improving interoperability through adherence to standards such as the BIRADS criteria for labelling and annotation, and a consistent file format, could markedly improve access and use of larger amounts of standardized data. This, in turn, could be increased further by GAN-based synthetic data generation, paving the way towards better health outcomes for breast cancer.

## Introduction

Each year, there are over 100 million mammograms taken around the world as part of the screening process for breast cancer. Each one of these mammograms requires at least one, and usually two reviews by specialist radiologists in order to identify anomalies and report on the mammographic image^1^. These requirements make breast cancer screening extremely expensive and resource intensive, and is the primary reason behind the fact that only 22 well-developed nations, such as Australia, the United Kingdom and New Zealand are in a position to offer government subsidised screening^2^.

With this in mind, the rates of breast cancer in emerging economies, such as India, Pakistan and Indonesia are growing at a rate of 0.5–1.5% each year, and with huge populations and limited financial resources, are unable to introduce traditional radiologist-led screening^2^. As a result, there is increased interest in the use of technology, notably deep learning and artificial intelligence, as a possible solution to this problem.

Producing such solutions however can be rather challenging, particularly when researchers initially turn to the possible sources of training data that exist in both the open-source and commercial realm. The evolution of scanner technology from predominantly film-based to digital has led to a large amount of quality disparity between the various datasets, as many are still based on digitized film scans (Digital Database for Screening Mammography (DDSM), Mammographic Image Analysis Society (MIAS) and the Breast Cancer Digital Repository Film (BCDR-F) dataset). Many are subject to either rigorous application procedures or a high financial barrier to entry (The Breast Cancer Digital Repository and OPTIMAM), and all differ considerably in terms of their chosen file formats, image size, labelling schema or volume.

These key areas of variance were addressed in 2016’s FAIR principles, which sought to provide a qualitative framework to understand datasets in view of their findability, accessibility, interoperability and reusability^3^. While the principles go some way towards understanding and promoting standards within scientific data, they do not provide a means to quantitatively rank and assess datasets. This has given rise to various systems, such as Bishop & Hank’s ranking criteria, which will be referred to throughout this paper^4^.

An important distinction to be aware of while applying the FAIR principles and quantitative systems such as Bishop & Hanks’, is that they are applied through the lens of a particular use case. In this paper, the appraisal and analysis will be based upon the author’s experience as the founder of a medical imaging startup company in finding and consuming mammographic data which is suitable to produce a generalisable tool that could localise and detect breast lesions in emerging economies. With this in mind, the author was actively seeking data which was precise enough to withstand regulatory scrutiny, and that which allowed them to produce technology that could operate with both modern digital scanners, and also the various film scanners that are common in various regions of the world.

This paper seeks to understand the various digital and film mammographic datasets that are available to researchers, both in the open-source and commercial realms. The individual strengths and weaknesses of each dataset will be appraised, along with their adherence to the FAIR principles and frameworks as viewed through the perspective of the author, who is working on a lesion detection approach. The seminal research on each dataset will be presented, and critically appraised. Finally, a discussion will pave the way towards potential advances and improvements to the landscape of mammographic data, including standardisation and synthetic data generation.

## Methods

A comprehensive search was conducted across reputable platforms, including ArXiV, Google Scholar, and the National Library of Medicine (NLM), to identify relevant papers and gather comprehensive information. These platforms were chosen for their extensive coverage of scientific literature and databases spanning various disciplines, including medical research.

The search was carried out over the period of one month, and was performed by the lead author along with review and input from the co-authors. The primary keywords that were used to guide the search were as follows:

1. Mammography Dataset(s)
2. Mammographic Dataset(s)
3. Breast Cancer Dataset(s)

Abstracts were chosen by the lead author based upon their adherence to the following inclusion criteria, and selected manuscripts were reviewed in detail by the lead author:

1. The dataset must contain only mammographic data.
2. The dataset must include some form of annotation schema relating to the images and/or the background tissue. These annotations could be whole-mammogram classifiers, bounding-boxes, elliptical or polygonal segmentation maps.
3. The dataset must contain a minimum of one hundred data points.
4. At least one published study on the use of the dataset on at least one of the aforementioned search platforms.
5. The original paper must have been cited at least five times, using Google Scholar’s citation tracking index.

In the upcoming section, a comprehensive evaluation will be conducted to assess the degree of compliance of each cornerstone dataset with the FAIR principles, which aim to promote data Findability, Accessibility, Interoperability, and Reusability. This evaluation will encompass both qualitative analysis based on the author’s startup experience and quantitative assessment using the FAIR framework established by Bishop and Hank^4^.

The qualitative analysis will take into account factors such as data availability, documentation quality, ease of access, and data sharing practices. This qualitative assessment will provide valuable insights into the practical aspects of using the datasets and their compliance with the FAIR principles.

In addition, a quantitative assessment will be conducted using Bishop & Hanks’ framework, which provides a structured methodology for evaluating the compliance of datasets with each aspect of the FAIR principles. This framework considers parameters such as metadata completeness, data accessibility through standardized protocols and interfaces, adherence to community standards and ontologies, and provision of persistent identifiers. By quantitatively analyzing these parameters for each dataset, a more objective assessment of their compliance with the FAIR principles can be obtained.

## Results

Through this process, a total of eight key datasets were identified, which are summarized in Tables 1, 2. Careful selection of these datasets was based on their relevance to the research topic and the availability of comprehensive mammographic imaging data within them. Each of these datasets offers valuable resources for the analysis and evaluation of breast imaging techniques. They exhibit variations in crucial aspects, providing a diverse range of characteristics for further investigation. These variations can be categorized into the following areas:

1. **Volume**: The number of mammograms included in each dataset varies, ranging from a few hundred to several thousand. This variation allows for studies with varying sample sizes and statistical power.
2. **Ground-truth labeling**: The nature of the labeling in the datasets varies. Some datasets have ground-truth labels based on biopsy or histology results, providing a higher level of accuracy and reliability. In contrast, others rely solely on doctors’ opinions, which may introduce subjectivity into the labeling process.
3. **Image source**: The datasets encompass mammograms sourced from different imaging modalities. Some datasets consist of mammograms acquired through digital sensors, known as digital mammography, while others include digitized screen film mammograms. This variation allows for investigating the impact of different imaging technologies on the performance of analysis algorithms.
4. **Image resolution**: The resolution at which the mammographic images were captured varies across the datasets. This variation affects the level of detail and image quality available for analysis.
5. **Image format**: The datasets include mammographic images in various formats, such as DICOM, TIFF, and JPG. Additionally, some datasets may undergo compression techniques to reduce storage requirements. The choice of format and any subsequent compression can impact the accessibility and quality of the images.
6. **Lesion labeling**: The datasets differ in how lesions are labeled. Some datasets provide labels for the entire mammogram, while others focus on specific lesions using bounding-box coordinates or polygonal segmentation maps. This variation allows for investigating the performance of algorithms in different lesion detection and localization scenarios.
7. **Background tissue descriptors**: The labeling schema in some datasets includes additional descriptors for background tissue characteristics, such as heterogeneity or density. These descriptors provide valuable contextual information that can be leveraged for analysis and classification tasks.
8. **Accessibility**: The datasets vary in terms of accessibility. Some datasets are freely available, allowing researchers to access and utilize them without any restrictions. Others may be open-source but subject to certain licensing terms, while a few datasets require a paid subscription or licensing agreement for access. This variation in accessibility can influence the availability and widespread use of the datasets in the research community.

**Table 1:** Summary of the available datasets, and their year of publication, image source, volume and license terms.

Name	Year	Image Source	Volume	Accessibility
DDSM	1996	Film	11560	Public
CBIS-DDSM	2017	Film	1644	Public
MIAS/Mini MIAS	1994	Film	322	Public
BCDR-F	2012	Film	3703	Public
BCDR-D	2012	Digital	3612	Public
INbreast	2011	Digital	410	Public
OPTIMAM	2020	Digital	100,000+	Commercial
CMMD	2022	Digital	3,728	Public

**Table 2:** Summary of the available datasets, with their image specifications and labelling schemas.

Name	Resolution	Image Format	Labelling	Segmentation	Background
DDSM^5^	42–50 Micron	LJPG	Mixed	Polygonal	None
CBIS-DDSM^7^	42–50 Micron	DICOM	Histological	Polygonal	None
MIAS/Mini	50 Micron	PGM	Unclear	Elliptical	Density
MIAS^10^					
BCDR-F^15^	1168 × 760px	TIFF (8-bit)	Mixed	Polygonal	None
BCDR-D^15^	3328 × 3560px	TIFF (16-bit)	Mixed	Polygonal	None
INbreast^18^	Raw/Full	DICOM	Histolgical	Polygonal	None
OPTIMAM^37^	Raw/Full	DICOM	Histological	Polygonal	None
CMMD^39^	Full	DICOM	Histological	None	None

### DDSM

The Digital Database for Screening Mammography (DDSM) is a popular large-scale mammographic dataset released in 1996^5,6^ The dataset contains 2,890 cases, including left and right cranio-caudal (CC) and mediolateral-oblique (MLO) views, for a total of 11,560 mammographic images. To date, due primarily to its open-source availability, age and popularity, the DDSM dataset has been cited in more than 80 distinct papers in mammographic machine learning and artificial intelligence.

Being a relatively old dataset, the DDSM library consists of scanned-film rather than digital mammographic studies, which have been scanned at a resolution of between 42 and 50 microns. The scanned images are also stored in a deprecated file format (LJPG) which requires the use of obsolete decompression code to access the data. This is perhaps the most significant general criticism of the DDSM dataset, justifying a score of 1/5 for interoperability in Bishop & Hank’s FAIR-ness framework^4^. The author found this compression technique to be challenging, and required the download and installation of software from Stanford University in order to access the underlying dataset.

The DDSM dataset categorises mammograms into four distinct ground-truth labels, and does not contain any molecular sub-type or background tissue descriptors:

- Malignant (914 Cases)
- Benign (870 Cases)
- Benign Without Callback (141 Cases)
- Normal (695 Cases)

Furthermore, the segmentation labelling of the dataset that seeks to delineate the margins of lesions within the mammograms, has been widely acknowledged to have inaccuracies^7^. This limits the ability of the DDSM dataset to be relied upon when developing tools that require very specific localisation or precision. In addition, the curators of the DDSM dataset clearly state that *malignant* cases are based upon a clinical gold-standard ground truth, such as core or post-surgical histopathological diagnosis, the other categories are less precise. For example, the *benign without callback* and *normal* labels are based upon the opinion of the radiologist interpreting the original mammogram. Furthermore, the ground-truth for the *benign* cases are highly ambiguous. According to the DDSM curators, benign cases can sometimes be based upon a histological outcome, but often are driven by a follow-up mammogram or ultrasound result alone. Data quality issues such as inaccurate segmentation and ambiguous labelling schemas fall under the *reusability* branch of the FAIR principles^3^. In the author’s experience, the quality limitations of DDSM were the primary reason that they chose not to include the repository within their overall training dataset, as it might introduce unnecessary skew and bias into the resultant models. Such sentiment is echoed by other researchers, who generally choose to use the DDSM dataset for experimental models in whole-mammogram classification, rather than precise localisation^8,9^.

In 2016, Levy and Jain trained both an AlexNet and GoogleNet convolutional neural network (CNN) binary classifier on data from the DDSM repository and compared the results to a radiologist^8,9^ The authors chose to split the dataset into *malignant* (therefore histologically confirmed as malignant) cases and *non-malignant*, which consisted of all other studies within the DDSM repository. Through applying this methodology, the authors were able to achieve a precision of 92.4% and a recall of 93.4% with their GoogleNet model, which on the face of it, is outperforming a human radiologist. However it is important to acknowledge that their model was trained and validated on data from the DDSM repository alone, which somewhat limits its generalisability to real-world cases, notably modern digital mammographic images. Furthermore, the bulk of the images that were labelled as *non-malignant* by the authors, are not histologically confirmed to be non-malignant, rather being based upon a radiologist’s opinion or imaging alone. This is likely to skew both the training and validation data within their approach.

### Mammographic image analysis society (MIAS) and mini-mias

The original MIAS repository includes a total of 322 scanned film mammograms at 50 micron resolution in Portable Gray Map (PGM) format and associated ground-truth data^10^. Elliptical segmentation of the masses are provided, and each region is labelled as either being malignant or benign. It remains unclear what the source is of the labelling in MIAS, and whether it relates to a biopsy or histology result, or is based upon a non-pathological finding such as the opinion of the radiologist. A unique feature of the MIAS dataset is that it offers descriptors of the radiological architecture of the mass and the heterogeneity of the background tissue. While the MIAS dataset is freely available for academic use, it is bound by certain copyright laws which may be a drawback for commercial ventures.

The same dataset was used to derive the Mini-MIAS dataset, which is essentially a downsampling of the original images to a 200 micron resolution, leading to images of 1024 × 1024 pixels^10^. This reduces the size of overall dataset somewhat, but leads to a reduction in the resolution of the images. The smaller download size of Mini-MIAS makes it popular with online competitions and bootstrapped approaches, although aside from this, it is difficult to make a case for it in academic research.

The achilles heel of both MIAS and Mini-MIAS is that the ground-truth labelling schema is based on the opinion of the radiologist rather than a histological outcome. Furthermore, the datasets adopt an elliptical segmentation format, which is less precise than polygonal annotations. In a similar vein to the original DDSM repository, the MIAS and Mini-MIAS datasets would score poorly on the interoperability and reusability metrics offered by the FAIR principles. This was also reflected in the experience of the author, who chose not to include these datasets due to these factors. One area where the MIAS dataset excels is the inclusion of background class descriptors, which have been extensively used in developing machine learning approaches for approximating breast density. The assessment of breast density is of great interest to reporting radiologists, as it is used as a clinical indicator in the well-known Breast Imaging and Reporting Data System (BIRADS)^11^.

A study by Muhimmah and Zwiggelaar in 2006^12^ demonstrated the combination of aggregate histogram analysis and support vector machine classifiers (SVMs) to predict the breast tissue density using the MIAS dataset. When their model was applied back to the original MIAS images, they were able to demonstrate a 77.57% agreement between their predictions and the original MIAS background descriptor. A similar approach was taken by Liasis et al in 2012, which yielded similar results^13^. The limitations of histogram analysis and support vector machines were addressed by a later paper in 2019 by Shi and colleagues^14^, who used a CNN based approach with a categorical cross entropy loss function on the MIAS dataset. The authors demonstrated a combined accuracy of 83.6% through using CNN based models rather than SVMs.

### Breast cancer digital repository (BCDR)

The Breast Cancer Digital Repository (BCDR) was proposed in 2012, as a means to collect, digitize and curate film mammograms from the Portuguese breast cancer screening service to drive future research^15^. As such, the original BCDR repository contains 3,703 digitised film mammograms, but is no longer actively maintained. Each individual image in the original BCDR repository (BCDR-F) is stored as an 8-bit TIFF file with a resolution of 1168 × 720 pixels. An interesting feature of the BCDR repository, is that the labelling conforms to the Breast Imaging Reporting and Data Systems (BIRADS) standards which are already well understood by radiologists and breast clinicians. Under the guidance of the FAIR principles, this reliance on a well-established industry standard would improve the potential of BCDR to become a highly interoperable dataset. However, BCDR has barriers to accessibility that would hinder general researchers and commercial organisations accessing the dataset. For example, the maintainers allow access only through a strict application process, which usually only grants access to academic institutions with a formal project plan and a non-commercial goal. In the FAIR-ness quantification framework offered by Bishop & Hank, these accessibility issues would cause BCDR to score a 2/5 on this metric, despite scoring highly on interoperability and findability.

Of the 3703 mammograms in the repository, the following demonstrates the weighting between the different BIRADS classifications:

- Mass (639)
- Microcalcification (341)
- Calcification (145)
- Distortion (168)

Polygonal segmentations are included for the above positive classes, but the authors state that only some of the positive findings are confirmed by histology, with the rest being based upon clinical opinion alone. At the time of the original paper, only 276 of the 1493 segmentations were based on a pathology result. This would unfortunately limit the reusability of BCDR, and would score it 3/4 on Bishop & Hank’s framework, despite the use of BIRADS. In addition, annotations are provided for lesions only, and the repository contains no background tissue metadata.

Despite the original BCDR-F repository lacking active maintenance, an ongoing side project called BCDR-D includes only full-field digital mammograms stored as 16-bit TIFF files at a larger 3328 × 2560 pixel resolution^16^. At the time of writing, BCDR-D contains over 3,600 images which also conform to the same BIRADS standards as the original BCDR-F library.

In the author’s experience, the positive findings from both BCDR-F and BCDR-D were of use within their’s final training dataset. As these scans conformed to the quality and re-useability criteria that were fundamental to their commercial efforts, they were deemed appropriate for inclusion. Of note however, the author did find the application process to gain access to the dataset to be time consuming, taking approximately two months to be provided clearance, which may present a barrier to entry to smaller research teams.

A 2018 study by Chougrad et al utilised the BCDR dataset to train a CNN binary classifier which predicts *mass* and *non-mass* cases, and demonstrated a combined accuracy of 96.67% when evaluating on a subset of BCDR data^17^. The authors also applied an aforementioned mapping, and noted that when combining and curating data from BCDR alongside other sources, notably DDSM and INbreast, they were able to achieve a combined accuracy of 98.23% when evaluating on the MIAS dataset.

### INbreast

The INbreast dataset is a full-field digital mammographic repository made public by the Hospital de São João, in Porto, Portugal in 2011^18^. The dataset contains a total of 410 full-resolution digital mammographic images (as DICOM files) with polygonal segmentation within a separate XML file in OSIRIX format. INbreast contains four distinct classes: mass, calcifications, asymmetries and distortions, with no background tissue descriptors. Furthermore, all of the pathologies contained within INbreast are confirmed histologically, via a core biopsy or post-surgical specimen assessment. Through the focus on modern digital mammography and high-resolution resultant images, and curating and ensuring the accuracy of the annotations, INbreast has often been credited as the most reliable and precise open-source mammographic dataset^19^. The authors of INbreast have not provided any specific licensing terms, and have made the dataset freely available to researchers and commercial vendors.

On the face of it, INbreast is a wholly adherent dataset by the FAIR principles, however the small size of the repository limits its potential as anything but either an adjunct or validation set, which has been the case in numerous studies on machine learning in mammography^20–22^.

The author has used INbreast as part of their validation set, mainly on the basis of the precision of the data. Another interesting academic use of INbreast is in the creation of synthetic data using augmentation or Generative Adversarial Networks (GANs). In 2019, Zhang and colleagues used INbreast to train a Generative Adversarial Network (GAN) to generate synthetic data that could be used to supplement or train larger-scale deep learning approaches^22^. Furthermore, Huang et al in 2020 demonstrated that using aggressive augmentation, they were able to generate 7,632 images from the original 410 from the INbreast dataset^23^.

### Curated breast imaging subset of DDSM

#### (CBIS-DDSM)

Many of the criticisms of the original DDSM dataset were addressed in 2017 by Lee and colleagues^7,24,25^ when an updated and curated subset of the DDSM database was proposed. This dataset, known as the Curated Breast Imaging Subset of DDSM (CBIS-DDSM) implemented the following improvements:

- Filtering of the original dataset and removing images identified as technically low-quality
- Re-annotating the polygonal regions of interest where the original annotation was deemed inaccurate
- Re-categorising the data into two distinct groupings - *malignant* and *calcification* - thus removing all of the spurious ground truth labelling associated with the *normal*, *benign* and *benign without callback* cases.
- Decompressing the original LJPG files and upscaling them into 16-bit grayscale TIFF images
- Extracting the metadata from the accompanying CSV files, and bundling the 16-bit images and metadata into Digital Imaging and Communications in Medicine (DICOM) files.

The resulting CBIS-DDSM dataset contains 753 calcification cases, and 891 mass cases, all of which are encapsulated in industry-standard DICOM files, with a corresponding file that contains accurate polygonal annotations and ground-truth labelling for each region of interest. Based upon these improvements, the CBIS-DDSM improves considerably upon the FAIR-ness attributes of both interoperability and reusability. As a result, the CBIS-DDSM dataset became useable by the author, as the accurate scanned-film captures added much needed analog images into an otherwise digital training dataset. This was important as the author’s start-up were creating a generalisable tool, that could potentially improve patient outcomes in areas of the world where film scanners are still common, such as India and Pakistan. One of the primary criticisms of CBIS-DDSM is that the adherence to quality has led to a significant downsizing of the overall dataset, which generally requires it to be combined with other datasets^26^. However it remains a highly findable, accessible, reuseable and interoperable open-source dataset that has a number of applications, particularly within film mammogram segmentation and localisation.

As a follow-up to the criticisms of the work of Levy and Jain^8^, Agarwal and colleagues^27^ trained a similar CNN-based binary classifier using the CBIS-DDSM dataset rather than the original DDSM repository. In this study, three distinct CNN models were trained, VGG-16, ResNet50 and InceptionV3 with data categorised as containing either a *mass* or *non mass*^28–30^ In further contrast to Levy and Jain, the authors also chose to use a different digital mammography based dataset (INbreast) to validate the model upon. Their best results were obtained by applying transfer learning on an ImageNet pre-trained variant of InceptionV3, which yielded an aggregate validation accuracy of 84.16%.

Later research by Falconi and colleagues^31^ and Shen and colleagues^32^ sought to improve upon the results of Agarwal and colleagues by using CBIS-DDSM to train a variety of different CNN architectures. Ultimately however, their results failed to demonstrate tangible improvements, and varied markedly from the original paper in their curation and validation methodologies.

The improvements to the polygonal segmentation in CBIS-DDSM led to a 2020 study by Ahmed and colleagues^33^ training both a semantic segmentation (DeepLab) and instance segmentation (Mask-RCNN) architecture^34,35^ using it. Through applying a methodology known as *transfer learning*, which uses certain pre-trained features including edges and contours, they were able to achieve a mean average precision (mAP) of 80% and 85% with Mask-RCNN and DeepLab respectively, which is roughly equivalent to the performance of a radiologist^36^.

### OPTIMAM

The OPTIMAM dataset is a large-scale digital mammographic image database which can be licensed from the Cancer Research UK (CRUK)^37^. At the time of writing, the dataset contains approximately 20,000 full-resolution biopsy confirmed segmented cancer images, and 2400 biopsy confirmed segmented benign images. There are also approximately 6,000 histologically confirmed masses that have not yet been segmented by the annotation team, and approximately 40,000 normal breast images. OPTIMAM does not contain any background tissue descriptors. The OPTIMAM dataset is currently being maintained and developed, and licensees are able to obtain updated data from the CRUK as it becomes available.

Being a wholly histologically-confirmed digital mammography dataset, some comparisons are often drawn between OPTIMAM and the INbreast dataset. However with close to 100,000 DICOM-encoded mammographic images contained within OPTIMAM, it is significantly larger than INbreast (410). This has enabled a number of breakthroughs in mammographic machine learning, the most famous of which was published by Google’s AI subsidiary, Deepmind in 2020^38^. In their research, Deepmind utilised the OPTIMAM dataset and supplemented it with a small amount of additional data from screening in the USA. They then validated it against radiologists in both the UK and the USA and demonstrated the following improvements:

- An absolute reduction in false positives of between 1.2% and 5.7%.
- An absolute reduction in false negatives of between 2.7% and 9.4%.
- An overall AUC-ROC improvement of 12.1%.

An interesting point that relates to both the OPTIMAM dataset and the work of DeepMind, is that it is the first research project to outperform a human radiologist, albeit without proof through robust clinical trials and regulatory audits. This highlights the importance of both quality and volume in datasets, and the potential of artificial intelligence.

Clearly the OPTIMAM dataset would score highly for FAIR-ness in view of the high degree of interoperability through the use of DICOM, and re-useability through the adherence to histological labelling and high-quality annotation. Furthermore, it would score 4/4 for findability on the Bishop & Hanks framework, based upon the popularity of the dataset, and the use of easy to navigate metadata. However, the moot point for the OPTIMAM dataset is the accessibility of the dataset, primarily on the grounds of the lengthy application process, and commercial licensing terms that can be expensive both in the short and medium term. The licensing and application process would render the OPTIMAM dataset a score of 1/5 for accessibility under Bishop & Hanks’ framework.

The alternative viewpoint on the accessibility of the OPTIMAM dataset presents a double-edged sword. Curating and maintaining a dataset of this quality and scale would require a dedicated team of people, and it would likely not be possible to provide this dataset without some form of commercial terms. Without licensing fees, the quality of the overall dataset would suffer markedly. As a start-up enterprise, the licensing fees did present a barrier to the author’s start-up company, who were required to raise a significant amount of capital to gain access to OPTIMAM. Clearly this would present a non-negotiable barrier to smaller research teams, which is likely the reason that OPTIMAM is generally consumed by commercial (or commerce-backed) teams.

### The chinese mammography database(CMMD)

The recently published Chinese Mammography Database (CMMD) comprises a total of 3,728 digital studies divided into two sub-datasets: CMMD1 and CMMD2^39^. Each sub-dataset consists of full-resolution DICOM files accompanied by histologically-confirmed outcome data. One notable feature that sets CMMD apart from previous datasets is the inclusion of molecular subtype information within the CMMD2 subset. This valuable information holds great potential for researchers investigating the relationship between phenotypes and precision medicine. Both CMMD subsets are readily accessible under the Creative Commons (CC BY 4.0) license, and can be easily downloaded via a self-hosted link provided by the authors. However, a potential limitation of the CMMD dataset is the absence of annotations indicating the region of interest within the mammogram, which restricts its applicability for researchers working on lesion detection techniques. Furthermore, the CMMD repository does not contain any information pertaining to the background tissue.

Due to this limitation and its relatively recent release, there have been limited studies examining the performance of the CMMD dataset on real-world clinical cases. In March 2023, Boudouh *et al*. published a paper analyzing the impact of fine-tuning various well-known convolutional neural networks using a combined dataset of CMMD, Mini-MIAS, and DDSM^40^. Their results demonstrated that their approach achieved an accuracy ranging from 99.54% to 99.90% in a whole-mammogram classification task, utilizing the InceptionV3 architecture.

On the surface, the CMMD dataset aligns well with Bishop and Hanks’ framework, showcasing its inherent FAIR principles. The ease of sourcing and access, coupled with interoperable and reusable images and descriptors, positions CMMD as an invaluable resource for mammographic researchers, especially those focusing on the Chinese demographic or those seeking to combine CMMD with related datasets. However, it is important to note the lack of bounding-box or polygonal segmentation annotations as a primary drawback, limiting its utility primarily to whole-mammogram classification or possibly within the domain of synthetic data generation.

## Discussion

**Fig. 1:**
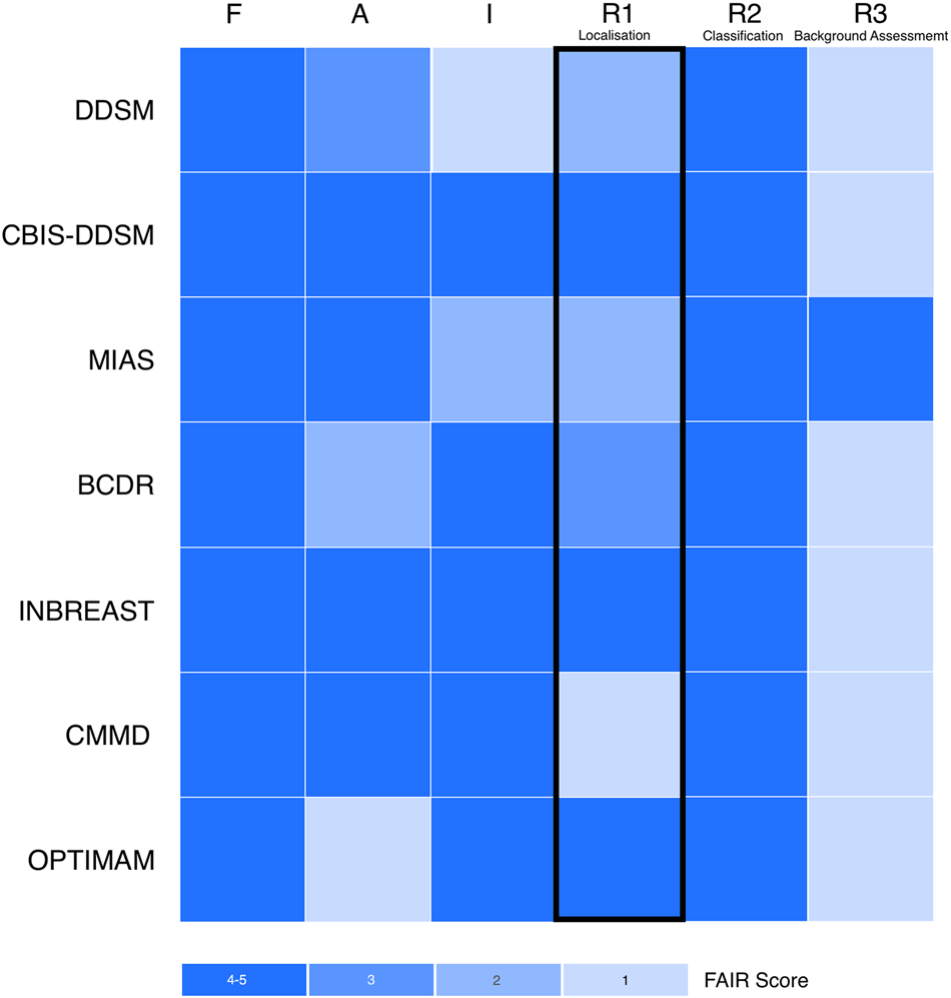
Heatmap visualisation of the FAIR-ness descriptors applied to the various available mammographic datasets, with R (reusability) score given for the primary use cases of lesion localisation, whole-mammogram classification and background tissue assessment^4,43^.

There are several differences between the available datasets for mammographic imaging researchers, which mainly relate to dataset volume, quality and type of labeling schema, whether the images are film or digital, and the licensing terms. Each of these differences generate a landscape of poor interoperability between existing datasets, which fundamentally provides context to the slow progress made by research teams in developing generalisable and clinically validated tools for mammographic imaging. A heatmap overview of the various datasets is provided in Fig. 1, which highlights the core differences in view of the four dimensions of data FAIRness. The heatmap also provides a breakdown of the reusability dimension into each individual machine learning use-case, notably localisation tasks, classification tasks and background assessment applications.

Prior to going into further detail on potential improvements and standards that can be employed by future data contributors, it is important to highlight the degree of different possible clinical use-cases for mammographic imaging, and that producing good mammographic imaging data is not a one-size-fits-all approach.

### Clinical use cases

As alluded to earlier, certain mammographic datasets exhibit features that make them more suitable to a particular application. This is not something that is considered by the FAIR principles, nor should it be, but it is an important factor in the decision making process when determining which datasets would be a good fit for a particular research project.

The poor scores for interoperability and reusability exhibited by the MIAS dataset, were essentially viewed through the lens of the author, who was developing a tool leveled at localisation and detection of lesions within a mammogram. Given that MIAS is a more appropriate dataset for background tissue assessment, it was deemed non-standard and too poorly annotated for the author’s use. However, for a research team focusing on breast density assessment, MIAS would score far better.

Furthermore, research teams that are looking to develop whole-mammogram triaging systems, that produce a binary outcome, such as malignant or non-malignant, could extract significant value from the CMMD dataset. Furthermore, the CMMD dataset may be extremely useful for researchers working on precision medical image analysis systems whereby the molecular subtype is of importance. The DDSM dataset may also be valuable to whole-mammogram tasks, due to the fact that the underlying malignant classification schema is deemed to be accurate.

**Fig. 2:**
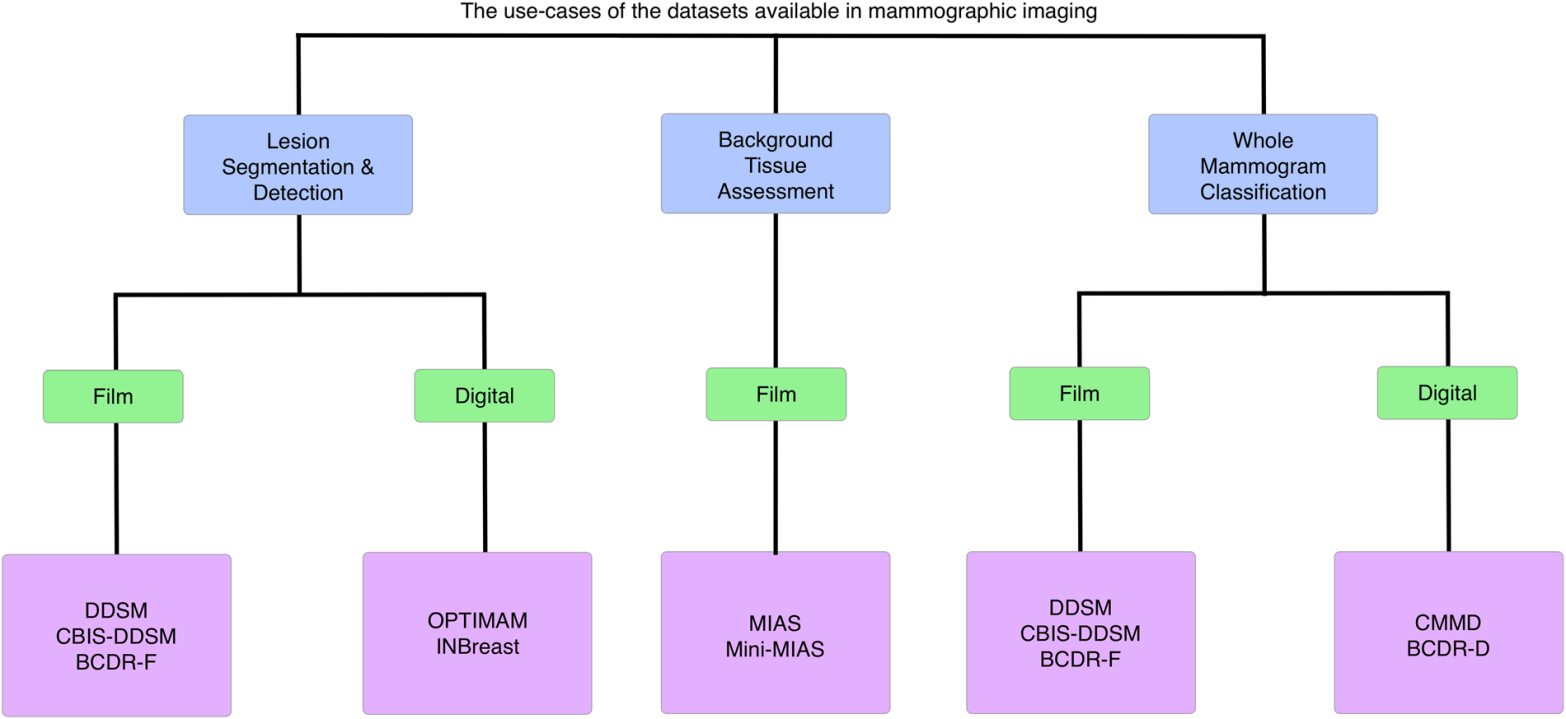
A comparison of clinical use cases.

Suffice to say, the FAIR principles, and the quantification of them, is a subjective endeavour that is subject to the requirements of the particular researcher. An overview of the decision making process, and the suitability of certain datasets is provided in Fig. 2.

### Film and digital scans

Another key differentiator in the available mammographic datasets, is the use of film and digital images. This is a highly important consideration, as the output of a scanned film mammogram is markedly different to the direct capture of a modern digital mammogram. The majority of current research is focused on digital mammography, as such scanners have largely displaced film scanners in the developed world. However, the incidence of breast cancer is increasing at a rate of 0.5–1% every year in developing regions, where film scanners are still the de-facto means by which mammograms can be taken^2^.

For commercial ventures, such as the author’s own medical imaging company, who are seeking to reduce the reliance on radiologists as part of the mammographic pipeline, and thus make screening possible in developing economies, the inclusion of film mammograms into the training dataset is mandatory. Therefore, film-based repositories such as CBIS-DDSM BCDR-F are highly important.

### Interoperability and standardisation

One of the key areas in which the available mammographic datasets vary, is their ability to interoperate with each other. In clinical medicine, the BIRADS standard of identifying and localising a lesion is generally considered to be the gold standard^41^. However, the majority of the datasets (aside from MIAS) do not follow this standard, rather they adopt more intuitive classification schemas, such as DDSM’s *malignant* and *benign* labelling. This ultimately leads to datasets becoming more interoperable with each other if they *choose not* to follow the clinical standard.

In addition to labelling, the image formats adopted by the individual datasets vary considerably. For example, the original DDSM team chose to use LJPG, MIAS uses PGM files, whereas OPTIMAM provides DICOM files. A possible solution to standardising these approaches would be choose a single lossless image format, such as TIFF. TIFF could encompass the full 16-bit DICOM data captured by the high resolution scanners in OPTIMAM, and also allow the smaller Mini-MIAS images to be upscaled to a standard size.

### Dataset volume

The FAIR principles consider datasets on the grounds of their findability, accessibility, interoperability and reusability. They do not consider the volume of the dataset to be a factor, although obviously it is important to machine learning researchers. For example, the INbreast and OPTIMAM datasets are intrinsically similar, although due to the small size of INbreast, it has limited utility in the commercial world. OPTIMAM on the other hand, with over 100,000 mammograms, has enabled teams such as DeepMind to produce models that can surpass the performance of a radiologist for the first time in over fifty years of research.

### Synthetic data generation

The role of synthetic data generation, or using machines to create artificial yet useful data, has been widely studied and has demonstrated promise in other areas of deep learning, such as autonomous vehicles^42^. As discussed previously, Huang *et al*. demonstrated that applying a Generative Adversarial Network approach to the relatively small INbreast dataset was able to generate over 7,000 unique mammograms^23^.

At the time of writing, there are relatively few datasets available to researchers, and even fewer that are free and open-source, which contain validated biopsy-proven labelling schemas (BIRADS category 6). In order for deep learning to consistently exceed the performance of a human radiologist, far more histologically confirmed data from diverse sources is clearly a prerequisite. The work by Huang et.al demonstrates the potential of using technology to expand the size of small datasets such as INbreast, but what if this approach was taken on the entire corpus of OPTIMAM’s dataset? Theoretically, the 20,000 mammograms within that dataset could yield over 350,000 histologically confirmed malignancies, and 42,000 benign lesions.

Such an approach could vastly increase the volume of the data that is available to researchers today, and enable the creation of far more accurate technology, albeit without the degree of generalisability that would be required for solving the issues in emerging economies.

### Supplementary information

Quantisation framework taken from Bishop, Bradley Wade, and Carolyn Hank.

## Data Availability

The DDSM dataset is available at http://www.eng.usf.edu/cvprg/mammography/database.html, and contains linearly numbered chunks of data between 2GB and 7GB for each of the cancer, benign, normal and benign without callback categories. The DDSM website also contains source code and information on how to utilise the data which is archived in LJPG format, and also provides a tool http://www.eng.usf.edu/cvprg/mammography/DDSM/software/software_v1.1.tar.Z. The CBIS-DDSM dataset is currently hosted on Kaggle, and is available at https://www.kaggle.com/datasets/awsaf49/cbis-ddsm-breast-cancer-image-dataset. The data can be downloaded using Kaggle’s command line interface, or manually through the website. The data is grouped into labels within the CSV folder, and images within the JPG folder. The original and full MIAS repository is available from https://www.repository.cam.ac.uk/handle/1810/250394 and the downsampled Mini-MIAS is available from http://peipa.essex.ac.uk/info/mias.html. The images contained within MIAS and Mini-MIAS are encoded with the PGM format, which is generally useable on modern Windows, Linux and Macintosh operating systems, or is fully supported by the open-source GIMP editor, which is available from https://www.gimp.org. The BCDR dataset, both digital and film, is available through registration and application at https://bcdr.eu. While the original repository for the INbreast dataset is unavailable, the data is freely available on Kaggle https://www.kaggle.com/datasets/ramanathansp20/inbreast-dataset. The dataset contains original DICOM images, along with corresponding medical report data and polygonal annotations. The CMMD dataset is freely available for download and attribution through the Creative Commons license from https://wiki.cancerimagingarchive.net/pages/viewpage.action?pageId=70230508 Being a commercially licensed dataset, the OPTIMAM dataset is available only to approved research teams under a license agreement. Applications to access can be made through the Cancer Research United Kingdom (CRUK) at https://www.cancerresearchhorizons.com/licensing-opportunities/optimam-mammography-image-database-omi-db Figure 1 was generated through the application of Bishop & Hank’s FAIRness framework to each indidvidual dataset. Figure headers relate to the Findability, Accessibility and Reuseability metrics on a scale of 1–4, and Interoperabilty on a scale of 1–3. These values were assessed and normalised into a scale of 1–5 in Fig. 1, in addition to the inclusion of three distinct reuseability dimensions for three clinical use cases - lesion localisation, whole-mammogram classification and background tissue assessment. The data used to construct Fig. 1 is available in CSV format at 10.6084/m9.figshare.23732889^43^

## Code availability

No custom code was used in this research article.
